# Nuclear face of Tau: an inside player in neurodegeneration

**DOI:** 10.1186/s40478-023-01702-x

**Published:** 2023-12-12

**Authors:** Neelam Younas, Tayyaba Saleem, Abrar Younas, Inga Zerr

**Affiliations:** 1 University Medical Center Göttingen, National Reference Center for Surveillance of TSE, Department of Neurology, Robert-Koch strasse 40, 37075 Göttingen, Germany; 2 German Center for Neurodegenerative Diseases (DZNE), Göttingen, 37075 Germany

**Keywords:** Nuclear Tau, Alzheimer’s disease, Nucleolus, Nuclear lamina, Tau isoforms

## Abstract

Tau (Tubulin associated unit) protein is a major hallmark of Alzheimer’s disease (AD) and tauopathies. Tau is predominantly an axonal protein with a crucial role in the stabilization and dynamics of the microtubules. Since the discovery of Tau protein in 1975, research efforts were concentrated on the pathophysiological role of Tau protein in the context of the microtubules. Although, for more than three decades, different localizations of Tau protein have been discovered e.g., in the nuclear compartments. Discovery of the role of Tau protein in various cellular compartments especially in the nucleus opens up a new fold of complexity in tauopathies. Data from cellular models, animal models, and the human brain indicate that nuclear Tau is crucial for genome stability and to cope with cellular distress. Moreover, it’s nature of nuclear translocation, its interactions with the nuclear DNA/RNA and proteins suggest it could play multiple roles in the nucleus. To comprehend Tau pathophysiology and efficient Tau-based therapies, there is an urgent need to understand whole repertoire of Tau species (nuclear and cytoplasmic) and their functional relevance. To complete the map of Tau repertoire, understanding of various species of Tau in the nucleus and cytoplasm, identification if specific transcripts of Tau, isoforms and post-translational modifications could foretell Tau’s localizations and functions, and how they are modified in neurodegenerative diseases like AD, is urgently required. In this review, we explore the nuclear face of Tau protein, its nuclear localizations and functions and its linkage with Alzheimer’s disease.

## Background

Tau protein is a member of microtubule-associated proteins (MAPs) family that are crucial modulators of microtubule dynamics in the cell [1, 2]. In the brain, Tau is a major regulator of neuronal microtubules assembly and stabilization required for morphogenesis and axonal transport. It is also expressed in glial cells [3] and in non-neural cells, such as fibroblasts and lymphocytes [4, 5].

Tau protein is mainly localized in axons in the mature neurons, but other cellular localizations of Tau protein has also been reported e.g., in the nucleus (nucleolus) [6], mitochondria [7], at the plasma membrane [8], soma [9], dendrites [10], synapses [11] and extracellular vesicles [12]. This vast localization pattern of Tau protein suggests that in addition to its main function as a regulator of microtubule dynamics, it has diverse pathophysiological roles in the cell [6, 13].

Tau, a natively unfolded protein, largely soluble and exhibits a very less tendency for aggregation [14].Tau protein is encoded by *MAPT*, comprising of 16 exons located on 17q21 chromosome location [15]. In the human brain, Tau has six main isoforms which are generated by alternative splicing of exons 2, 3, and 10 [1, 16]. Tau isoforms vary depending on the number of N-terminal 29-residue inserts and presence or absence of microtubule binding domain (R2) [16]. Tau isoforms vary in terms of expression of R domains (R1, R2, R3, and R4) consisting of 31–32 distinct and similar amino acid motifs. Isoforms that express the R2 domain are known as 4R while those lacking the R2 are designated as 3R. Primarily, Tau isoforms are denoted as 0N3R, 0N4R, 1N3R, 1N4R, 2N3R, 2N4R, however they are also known by the residue number or the clone name [1, 17].

Since the discovery of Tau protein, research efforts were concentrated on it’s role in the context of microtubules, even though, multiple localizations of Tau protein have been reported [18]. Tau is highly abundant in neuronal axons [19], but under various pathophysiological conditions, it can also be found in the soma [9], the dendrites [10], and the nucleus [10, 20].

Subcellular fractionation of murine brain tissue has been performed to identify distinct localization patterns for Tau isoforms [21]. The isoform 0 N is enriched in cell bodies and axons with a slight staining in nuclei and dendrites. The isoform 1 N is predominantly detected in the soluble nuclear fraction (neuronal nuclei). It is also detectable in cell bodies and dendrites, but not in axons. The isoform 2 N is highly expressed in cell bodies and axons, with a detectable expression in dendrites and a very low signal in nuclei [21]. This data indicate significant differences in the expression of Tau isoforms in the murine brain that likely reflect different neuronal functions. However, the localization pattern of nuclear Tau is different between human and murine cell-types [22, 23], which might be the one reason that transgenic mice models do not recapitulate full spectrum of AD pathology. Interestingly, these murine Tau isoforms do not contain any nuclear localization signal (NLS) for their transport from the cytoplasmic to the nuclear compartment [21]. Furthermore, Tau exists in different conformations in the nucleus, and specific epitopes are accessible to different antibodies in a compartment-dependent manner [23]. Understanding of complete spectrum of nuclear Tau species and their mode of nucleocytoplasmic transport may shed light on the nuclear face of Tau under pathophysiological conditions.

The multiple localizations of Tau protein suggest condition- and subcellular microenvironment-dependent interactions of Tau protein [24, 25] with different subcellular compartments e.g., nucleus. This suggests that Tau is a multifunctional protein and its role in pathophysiology of the neurons needs to be regularly reviewed in the light of emerging discoveries. Here, we discuss the nuclear face of Tau at different levels starting from nuclear lamina down to the nucleolus, and how its nuclear face contributes to neurodegeneration in Alzheimer’s disease.

### Nuclear lamina and Tau

Tau is a multifunctional protein and precise function of which depends on its localization. The localization of Tau protein to the inner side of the nuclear lamina [26, 27] and regulation of nuclear pore complex emphasizes a crucial role of Tau [28, 29] protein in maintaining nucleus integrity. The oligomeric form of Tau directly binds to lamin B receptor and lamin proteins, which lose their solubility upon oligomerization and also their nuclear membrane localization, ultimately leading to disruption of the nucleocytoplasmic interface [30]. Tau regulates nuclear Lamin B1 expression [31, 32]. Tau-dependent reduction of Lamin B1 leads to disruption of nuclear lamina [33]. The expression of full-length Tau or its truncated variant (Asp421-truncated Tau) in SH-SY5Y cells leads to formation of nuclear envelope indentations [34].

In Huntington’s disease and frontotemporal dementia, nuclear envelope indentations are filled with rod-like Tau deposits [35–37]. Likewise, Transgenic mice expressing mutant P301L-Tau shows disruption of NL [27]. One of the deleterious consequences of disruption of nuclear lamina is impairment of nucleocytoplasmic transport, which has been related to both overexpression of Tau and pathological Tau [27, 32, 38, 39]. The first indication of nuclear Tau in Alzheimer’s disease came from transmission electron microscopy (short paired helical filaments) in frontal lobe of AD cases [40]. Alterations in nuclear lamina are a characteristic of aging [41]. Indeed, mutations in the lamin A/C protein leads to the ‘’accelerated aging disorder Hutchinson-Gilford progeria syndrome’’ [42]. Alterations in nuclear lamina have been reported in AD brains [28, 32], where neurons exhibit a more complex structure of nuclear lamina.

Modifications in the nuclear lamina can initiate alterations in the DNA organization, through modification of nucleocytoplasmic transport. Iinteractions of Tau protein with the nuclear envelope contribute to defects in RNA and protein nucleocytoplasmic transport [27, 43]. Controlled nucleocytoplasmic exchange of cellular biomolecules—such as mRNA and rRNA, transcription regulators, nuclear and cytoplasmic proteins—is crucial for key functions of cell survival e.g., stress response, signal transduction and proteostasis [44–46]. How alterations in the nuclear lamina contribute to all these crucial processes (directly or indirectly) under health and disease require further investigations.

### Nuclear speckles and Tau

Nuclear speckles (NSs) are membrane less organelles, which are sites of splicing factor storage and modifications, and are closely linked with RNA metabolism [47]. Nuclear Tau aggregates colocalize with nuclear speckles, and alter their composition and dynamics, in cellular models and in mouse brains [48]. In Alzheimer’s disease, pathological Tau drives ectopic accumulation of SRRM2, a core scaffold protein of nuclear speckles [49]. This depletion of SRRM2 may lead to altered splicing, transcription and translation, ultimately affecting neuronal physiology [50]. Two other nuclear speckles proteins MSUT2 and PABPN1 are also depleted from nucleus in cases of severe Alzheimer’s disease [51]. Furthermore, It has been reported that Tau aggregates particularly grow with endogenous mitotic interchromatin granules and cytoplasmic speckles [52] containing SRRM2 and PNN proteins. These evidence support the hijacking hypothesis for pathological-Tau in association with RNA to deplete critical workers from the nucleus (nuclear factors), affecting the biology of RNA in the nucleus. A similar sequestration of RNA binding proteins (e.g. TDP-43 and FUS) in cytosolic condensates, in amyotrophic lateral sclerosis has been associated with impaired nuclear RNA-processing [53–55]. Thus, the sequestration of RNA binding proteins and RNAs into pathological aggregates may signify a common pathophysiological feature in multiple neurodegenerative disorders affecting diverse cell types, with the depletion of crucial RNA processing factors from the nucleus, altering RNA processing, ultimately leading to altered gene expression. The identification of exact mechanism of speckle hijacking could highlight important aspects of Tau pathophysiology. Rescuing the SRRM2 splicing function by restoring its nuclear localization in the presence of Tau aggregates may mitigate neurodegeneration. The disruption of deleterious cytosolic Tau-RNA condensates using bait RNAs may restore nuclear localization of important nuclear factors. Clearly, urgent investigations are required to explore these hypotheses and their utility for therapeutic interventions.

### Nucleolus and Tau

Nuclear studies have shown a crucial role of Tau protein in nucleolar structure conformation [4, 56]. Tau colocalizes with crucial nucleolar factors such as nucleolin, upstream binding transcription factor, and TIP5 in cellular models and human brain tissues [56, 57]. It has been found that nucleolar chaperons are reduced in different AD brain regions including nuclear Tau [58]. Tau enhances interactions of nuclear proteins like T cell intracellular antigen 1 (TIA1) with ribonucleoproteins, suggesting a role for it in rRNA gene metabolism [57]. The Tau protein localizes in the nucleolar organizer region (pericentromeric heterochromatin) and at the dense fibrillar regions as shown by immunofluorescence [22, 59, 60].

Tau can interact with both ribosomes and rRNA through its association with RNA binding proteins [61, 62]. In tauopathies, the interaction between Tau and ribosomes is pronounced [61], suggesting an impairment of its functioning. Translocation of phodphorylated-Tau into the nucleus results in nucleolar dispersion and p53-dependent apoptosis, both of which contributes to neurodegeneration [63].

Recently, non-phosphorylated Tau (at residues Ser 195, 198, 199 and 202) has been described as a bona fide nucleolar protein in neuronal cell lines and human brain tissue [57]. An association between Tau and TIP5 (a main player of heterochromatin stability and ribosomal DNA transcriptional repression) in SHSY5Y cells and human brain tissue [57] suggests a crucial role of Tau/TIP5 in the stabilization of repressive epigenetic marks on the ribosomal DNA. However, further investigations are required to study the precise role of Tau in nucleolar remodeling complex. Furthermore, nucleolar stress (induced by glutamate) redistributes nucleolar non-phosphorylated Tau in a similar way to other nucleolar factors e.g. fibrillarin, and induce a nuclear influx of phosphorylated Tau (Thr231) which shows a distinct localization from fibrillarin and nucleolar Tau [57]. These findings suggest that different species of Tau are present in the nucleus and play specific roles depending on type of cellular distress.

### DNA and Tau

In the nucleus, Tau is involved in DNA protection and chromosome stability [4, 57, 64]. In vitro studies indicate that binding of Tau protein with DNA can increase the melting temperature of DNA [65] and protect it against heat shock induced double strand breaks, providing an evidence that nuclear Tau (non-phosphorylated) is crucial to cope with early stress responses in continuous changing microenvironment of neurons [66]. Further research could investigate the molecular mechanisms underlying this protective function and its implications for neuronal resilience in the face of environmental stressors. Moreover, identifying pathways through which Tau interacts with heat shock response machinery can provide us with potentially novel targets for therapeutic interventions.

It has been shown that Tau knockout cortical neurons are more susceptible to heat stress-induced and hypothermia-induced DNA breakage as compared to their wild-type counterparts [67]. In addition to its function in DNA protection, it has been shown to play a role in the modulation of gene expression. Exploring the regulatory mechanisms by which Tau influences gene transcription, can shed light on its role in neuronal homeostasis and its potential involvement in neurodegenerative diseases. Indirectly, Tau could affect the gene transcription by some compensatory changes in the gene expression [67]. Investigating how the loss of Tau affects gene transcription and whether compensatory mechanisms exist could offer insights into the broader consequences of nuclear Tau dysfunction.

To date, 14 genes have been identified, for which transcription has been reported to be significantly increased after Tau depletion in quantitative real-time PCR and microarray analyses [68, 69]. Tau plays an important role as an epigenetic regulator of gene expression, an organizer of heterochromatin, a contributor to chromosomal stability, and processor or silencer of ribosomal RNA [69]. All these evidence suggest that Tau can regulate genomic functions in the nucleus. Considering the significant regulatory role of the nucleus in the maintenance of cellular homeostasis and the presence of Tau in the nucleus of the AD brain, we focused on its nuclear role specifically in Alzheimer’s disease.

Considering the substantial impact of nuclear processes on cellular balance and the observation of Tau in the brains of Alzheimer’s disease patients, it is plausible to propose that Tau’s nuclear functions have a distinctive role in AD. These functions could potentially underlie the alterations in gene expression, instability in chromosomal structure, and disruptions in protein synthesis seen in AD. A focused investigation into the precise implications of nuclear Tau in the context of AD may reveal valuable insights into the disease’s development and provide leads for therapeutic interventions.

### Nuclear Tau and stress granules

Emerging evidence indicate that Tau protein is a regulator of biology of RNA binding proteins [70]. Tau promotes stress granule development and regulate the interactome of TIA-1, a main component of stress granules [13]. However, which species of Tau (nuclear or other?) participate in stress granule formation, remains enigmatic. As, TIA-1 protein, a classical marker of stress granules, upon stress exit from the nucleus to initiate stress granule formation. Plausibly, nuclear Tau may also translocate to cytoplasm upon stress and contribute to stress granule formation, which has been reported as nidus of Tau misfolding [71, 72]. Investigation of precise species of Tau (nuclear or cytosolic) involved in stress granule formation may shed important light on dual role of nuclear Tau and its nidus of misfolding.

### Nuclear Tau and aging

The levels of different isoforms of Tau protein could change in different types of neurons during the development, aging or diseases (tauopathies) in mammals. In some diseases, there is a toxic gain of function of altered Tau, due to the hyperphosphorylation or aggregation [73, 74]. These phenotypic changes are mainly found in aging organisms. Aging is a risk factor for several neurodegenerative diseases like Alzheimer’s disease or Parkinson’s disease. With aging, there is an increase of neuronal vulnerability to oxidative damage, that could modify Tau protein [75, 76] facilitating its aggregation [77, 78]. An age-dependent accumulation of Tau aggregation has been reported in a *C. elegans* model [79]. Also during aging, mitochondrial changes in the brain occur [80]. Abnormal binding of a mitochondrial protein, DRP1 (involved in mitochondrial fission) to Tau protein promote neurodegeneration through mitochondrial dysfunction [81].

Previous studies have shown a decrease in soluble Tau with increased aging [82] in resected human brain tissue as well as in post-mortem brains [83]. On the contrary, phosphorylated Tau (Thr 212) increases during aging, in the nucleolus and pericentromeric heterochromatin of pyramidal neurons in the CA1 region, with the maximum accumulation in senescent cells [26]. However, this phosphorylated form of Tau decreases in AD, at its later stages [26]. Since Thr212 is a direct target of kinase GSK3β [84], and the activity of GSK3β increase during aging, and AD pathology [84–86], the increased nuclear phosphorylated Tau with aging may be attributed to the increase of GSK3β [6].

Elevated intron retention, an alternative splicing process in which introns remain within mature mRNA transcripts, has also been associated with aging brains and the development of Alzheimer’s disease [87–89]. Specifically, the retention of intron 11 results in the translation of a novel truncated Tau11i isoform. These Tau11i proteins aggregate in granular-like formations within the temporal lobes of AD patients. It is of significant importance to uncover the mechanisms governing these distinct splicing events, understand how Tau11i proteins contribute to the formation of pre-tangles, and elucidate their role in the pathogenesis of AD [90].

Tau can be secreted into the extracellular space and can be transferred to the neighboring cells [91] e.g. neurons, astrocytes, and microglia, in a prion-like fashion. Particularly, an age-dependent spread of Tau protein has been observed in mouse brain. Aged animals show enhanced spreading of Tau in the hippocampus and neighboring cortical regions with accumulation of prominent misfolded Tau in entorhinal cortex [92]. All these evidence indicate that alterations in Tau protein levels (proportions), post-translational modifications, and spreading occur with aging. Future investigations addressing the heterogeneity of Tau species and their PTMs with age, and under different stressful conditions could highlight important aspects of Tau pathophysiology in normal aging and tauopathies.

## Nuclear Tau in Alzheimer’s disease

Tau accumulation is a major pathological marker in several neurodegenerative diseases. Recently, it was found that hyper-acetylation of Tau at residue 174 (Tau-K174ac) increases its nuclear accumulation which is triggered by DNA damage signalling or SIRT6 shortage [69]. In Alzheimer’s disease, genomic instability has been reported, and SIRT6’s role in this context may have implications for disease progression. Targeting the acetylation of Tau at residue 174 or restoring SIRT6 levels could mitigate Tau accumulation and potentially offer therapeutic strategies for Alzheimer’s disease. Research may focus on how this acetylation event influences Tau pathology and developing interventions aimed at modulating these factors. Presence of higher nuclear Tau (Tau-K174ac), increased nucleolin and decreased SIRT6 levels have been reported in the AD cases [93]. The Tau-K174ac toxicity has been attributed to its nuclear accumulation and nucleolar dysfunction [93]. In a recent study, the role of nuclear Tau (AT8 epitope) has been reported in the onset of Alzheimer’s disease. There was decreased immunoreactivity in senile neurons, as compared to younger one^’^s [94]. Furthermore, the findings from this study suggest nuclear Tau’s involvement in the abnormal activation of cell cycle in differentiated cells [94].

Nulcear translocation of Tau occurs via importin-α/β pathway [29]. Hyperphosphorylated Tau in the nucleus interferes with important cellular processes such as nucleocytoplasmic transport and mislocalisation of nuclear factors, contributing to cell death [27, 29, 95]. Further investigation could explore the specific mechanisms through which Tau disrupts these processes and its implications in neurodegenerative diseases, such as Alzheimer’s disease.

It is also of great importance to consider that the role of nuclear Tau in neurodegenerative diseases could be through stress-dependent inhibition of nuclear Tau function because of aggregation and hyperphosphorylation [57, 96]. Research could examine how stress-related factors impact nuclear Tau’s functionality and whether this contributes to disease pathogenesis. Phosphorylation and aggregation of Tau protein are among the major post translational modifications responsible for the formation of paired helical filaments (PHFs) and neurofibrillary tangles (NFTs) [97]. The binding of hyperphosphorylated Tau to the DNA alters its conformation and integrity leading to nucleosomal disorganization and altered gene expression [98]. Interestingly a recent study demonstrated a significantly increased Ca^2+^ nuclear concentration with the hyperphosphorylation of Tau. This may propagate a self-perpetuating loop to cause neurodegeneration [99]. Phosphorylation and aggregation of Tau, leading to the formation of paired helical filaments and neurofibrillary tangles, could be a key mechanism through which Tau disrupts nuclear functions. Investigating the impact of hyperphosphorylated Tau on DNA conformation, nucleosomal organization, and gene expression could provide insights into its role in neurodegeneration.

Moreover, the role of Tau protein as a chromatin modifier in Alzheimer’s disease and aging has been discovered. As compared to the younger neurons the aged neurons have increased levels of AT100 (p-The212-Ser214) immunoreativity in the dentate gyrus and hippocampal CA1 region [26, 100]. As the age progresses, positivity zone increases in intensity and frequency specifically near the nuclear membrane and nucleolus [101]. As the AD progresses, depletion of nuclear Tau is more evident with maximum depletion at late stages. At the late AD stage, AT100 immunopositivity can exclusively be found in the neurofibrillary tangles (NFTs) [58]. At initial stages, Tau exits the neuronal nucleus completely [58, 102] and causes global chromatin relaxation which consequently leads to abnormal transcription of various heterochromatin genes and dysregulation of euchromatin gene [31, 103]. Research may delve into the molecular pathways linking Tau’s nuclear exit with chromatin alterations and the subsequent transcriptional dysregulation. This information can eventually provide a great insight into factors causing abnormal transcription of heterochromatin genes which could further be intervened to stop these abnormalities in terms of therapeutic interventions.

The ultimate transcriptional silencing leads to decondensation of heterochromatin at perinuclear and pericentromeric regions [26, 27]. With the AD progression, disrupted nucleo-cytoplasm transport results in the mislocalization of many nuclear proteins in the cytoplasm [27, 95]. Investigating the specific genes affected by this silencing and their role in AD pathogenesis can offer insights into disease mechanisms.

**Fig. 1:**
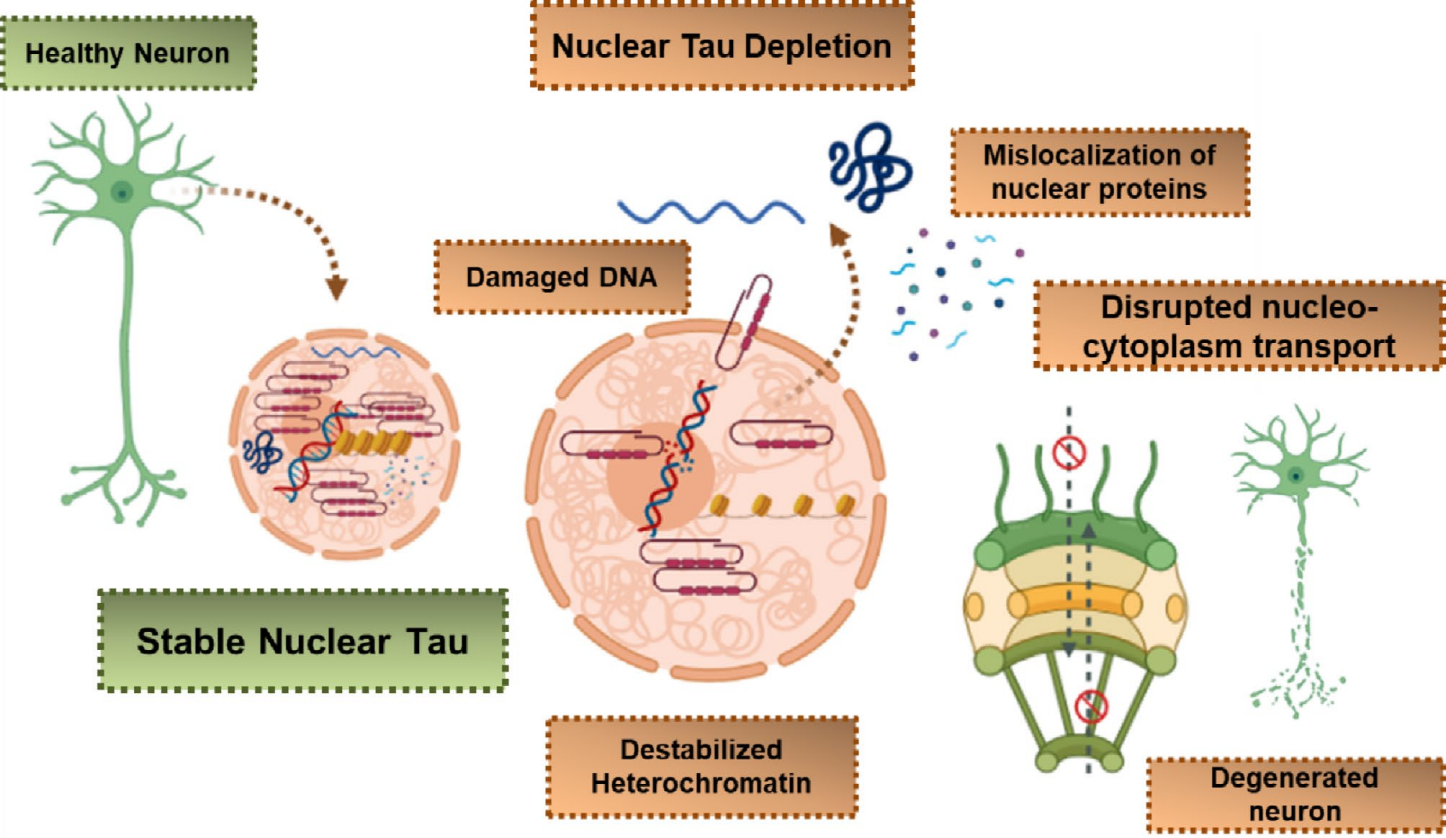
Role of nuclear Tau in Alzheimer’s disease. Nuclear Tau is involved in genome stability and maintenance of nucleo-cytoplasmic transport. Depletion of nuclear Tau leads to conformational changes in the heterochromatin and makes DNA vulnerable to damage, disturbs rRNA synthesis and ribonucleotide pool balance. The cytosolic Tau aggregates sequester nuclear factors leading to their depletion from the nucleus. Tau-mediated Impairment of nuclear pore complex disrupts nucleo-cytoplasmic transport, ultimately contributing to Tau-induced neurotoxicity (Created with https://www.BioRender.com)

The crucial role of Tau protein on the regulation of genome has been validated by microarray hybridization (ChiP-on-chip) assays and genome-wide immunoprecipitation (ChiP) [104]. Nuclear depletion of Tau contributes to an impairment of perinuclear heterochromatin in AD [26]. Exploring the mechanisms by which this impairment relates to AD-related epigenetic changes and its consequences on gene expression can provide us with valuable information for better disease interventions for diagnostics and therapeutics. A strong interaction of Tau with intergenic and intronic parts of DNA, coding for long noncoding RNA (lncRNA), has been reported [104]. Investigating the specific lncRNAs involved, their functions, and their role in the regulation of chromatin and gene expression may uncover novel pathways in AD pathogenesis. Tau and lncRNAs both regulate transcription of chromatin and gene expression indirectly, these processes are remarkably deregulated in AD [105, 106]. Nucleolar depletion of Tau disturbs the tRNA synthesis and destabilizes rDNA loci [64]. Altogether nuclear Tau is essential for transcriptional regulation, NAD’s stability, and regulated functioning of the nucleolus [26].The role of Tau in nuclear pore complex dysfunction points out its probable contribution towards Tau-induced neurotoxicity in AD and tauopathies. Investigating the mechanisms through which Tau disrupts nuclear pore complex function and its implications for cellular homeostasis can provide insights into disease progression. The nuclear functions of Tau and their disruption in AD is summarized in Fig. 1.

## Conclusions

Since the discovery of Tau protein in 1975, research efforts were concentrated on the role of Tau protein in pathophysiology in the context of the microtubules dynamics and stabilization, even though, for more than three decades, different localizations of Tau protein have been discovered e.g., in the nuclear compartments. Discovery of the role of Tau protein in various cellular compartments especially in the nucleus opens up a new fold of complexity in tauopathies. Perturbations in the Tau protein (as happens in AD and other tauopathies) could alter its multiple functions in the nucleus enhancing genome vulnerability and neurodegeneration. Its interaction with several nuclear components strongly suggests that it could play multiple functions in the nucleus, although more investigations are required to find out a precise role of Tau in these processes. Nuclear tau heckles nuclear speckles leading to RNA splicing defects, nucleo-cytoplasmic transport deficits, chromatin organization disturbances and nucleolar organization deficits. A through identification of different species of Tau (nuclear and cytosolic), their mode of nucleo-cytoplasmic transport, neuronal microenvironment-mediated post-translational modifications of Tau protein are crucial aspects to understand the whole repertoire of Tau and its nidus of aggregation. Furthermore, to find out effective Tau-based therapies, there is an urgent need to understand precise functional relevance of these diverse cellular localizations of Tau protein and how it is altered during neurodegeneration.

## Data Availability

Not applicable.

## Abbreviations

AD: Alzheimer’s disease
CSF: Cerebrospinal fluid
NFT: neurofibrillary tangles
NSs: nuclear speckles
MAPT: microtubule associated protein Tau
MAPs: microtubule-associated proteins
NL: Nuclear Lamina
PHF: paired helical filaments
p-Tau: phosphorylated Tau
Tau: tubulin associated unit
T-Tau: total Tau
